# ORVAL: a novel platform for the prediction and exploration of disease-causing oligogenic variant combinations

**DOI:** 10.1093/nar/gkz437

**Published:** 2019-05-31

**Authors:** Alexandre Renaux, Sofia Papadimitriou, Nassim Versbraegen, Charlotte Nachtegael, Simon Boutry, Ann Nowé, Guillaume Smits, Tom Lenaerts

**Affiliations:** 1 Interuniversity Institute of Bioinformatics in Brussels, Université Libre de Bruxelles-Vrije Universiteit Brussel, 1050 Brussels, Belgium; 2 Machine Learning Group, Université Libre de Bruxelles, 1050 Brussels, Belgium; 3 Artificial Intelligence lab, Vrije Universiteit Brussel, 1050 Brussels, Belgium; 4 Laboratory of Human Molecular Genetics, de Duve Institute, UCLouvain, 1200 Brussels, Belgium; 5 Hôpital Universitaire des Enfants Reine Fabiola, 1020 Brussels, Belgium; 6 Center of Human Genetics, Hôpital Erasme, 1070 Brussels, Belgium

## Abstract

A tremendous amount of DNA sequencing data is being produced around the world with the ambition to capture in more detail the mechanisms underlying human diseases. While numerous bioinformatics tools exist that allow the discovery of causal variants in Mendelian diseases, little to no support is provided to do the same for variant combinations, an essential task for the discovery of the causes of oligogenic diseases. ORVAL (the Oligogenic Resource for Variant AnaLysis), which is presented here, provides an answer to this problem by focusing on generating networks of candidate pathogenic variant combinations in gene pairs, as opposed to isolated variants in unique genes. This online platform integrates innovative machine learning methods for combinatorial variant pathogenicity prediction with visualization techniques, offering several interactive and exploratory tools, such as pathogenic gene and protein interaction networks, a ranking of pathogenic gene pairs, as well as visual mappings of the cellular location and pathway information. ORVAL is the first web-based exploration platform dedicated to identifying networks of candidate pathogenic variant combinations with the sole ambition to help in uncovering oligogenic causes for patients that cannot rely on the classical disease analysis tools. ORVAL is available at https://orval.ibsquare.be.

## INTRODUCTION

Massively parallel Next Generation Sequencing (NGS) has revolutionized the field of medical genetics, allowing the analysis of large cohorts and the identification of genomic variants that cause disease or modulate the severity or response to therapy (1,2). This vast amount of genetic data allowed for the emergence of Genome Wide Association Studies (GWAS) and linkage analyses methods, the creation of various variant-to-disease databases, as well as the development of bioinformatics and machine-learning tools for single variant pathogenicity prediction and prioritization (3–5).

Even though the emergence of such resources has greatly improved our understanding of Mendelian cases (i.e. the traditional view of one-to-one variant-phenotype association), many difficulties remain in identifying the causes of a large amount of human diseases due to phenotypic variability, disease heterogeneity and incomplete penetrance. These difficulties indicate the presence of more intricate genetic models that involve the interaction between several different variants and genes (6). Databases, as well as predictive and exploratory tools need to be improved to also deal with genetic models ranging from *digenic* or *oligogenic*, where a combination of causative variants is distributed among two or a small amount of genes respectively (7,8), to the *multi-factorial* or *complex* diseases, which are caused by a combination of genetic and environmental factors (6,9). Examples for this spectrum consist, on one end, of diseases like Bardet-Biedl syndrome (10,11) and cystic fibrosis (12) and, on the other end, neurodevelopmental disorders like intellectual disability and autism (13).

At the molecular level it is generally hypothesized that oligogenic diseases involve mutated proteins that are implicated in the same biological pathways or protein complexes (14). As a consequence, network-based approaches, which allow for the modelling of complex relationships between variants, genes and proteins, and offer a way to study diseases at a higher level of abstraction, quickly attracted attention (15). Protein-protein interaction (PPI) networks (13,16), disease networks (16,17) and gene expression networks (18) have all been used to assist in this direction. Yet, all these approaches start from a monogenic base, which may be incompatible for detecting oligogenic causes. Moreover, it was shown that such an approach may not perform better than simply linking genes randomly in a network (19). This problem may be overcome when predictive methods immediately identify the cross-gene pathogenic associations as opposed to first identifying single genes and then trying to link them using alternative sources of information.

This step has become feasible as the number of *digenic* or *bi-locus* cases reported in scientific literature reached a sufficient amount, resulting in the creation of the Digenic Diseases Database (DIDA) (20). All the pathogenic variant combinations in DIDA belong to one of three classes of *digenic effects* (DE): the *true digenic* class that requires the presence of variants in both genes to trigger the disease phenotype, the *modifier* class that involves a variant on the major gene that induces a disease phenotype and a variant in a modifier gene that alters the severity of symptoms or age of onset, and the *dual molecular diagnosis* class that involves the independent segregation of disease-causing monogenic variants in two different genes, leading independently to two different clinical diagnoses. We use here the terms *digenic* and *bi-locus* interchangeably, when referring to all the three DE classes mentioned above.

This resource allowed for the development of VarCoPP, an interpretable machine learning method able to predict candidate disease-causing bi-locus variant combinations (21). Furthermore, a predictor that identifies the DE class of a predicted digenic variant combination was also created (22). This information can be particularly useful in order to understand the effect of a variant combination when there is no pedigree genetic information available. These innovative methods may provide an important aid in identifying potential oligogenic disease signatures from patient data, which is exactly the purpose of the platform presented here.

ORVAL provides a novel user-friendly web platform that allows clinicians and researchers to predict the potential pathogenicity of an individual’s oligogenic variant combinations and examine candidate oligogenic signatures in the context of their pathways, PPIs and cellular locations. By identifying and supporting with biological evidence combinations of variants, needing further validation, this tool aims to become an important agent in exploring the relevant molecular and biological patterns underlying oligogenic diseases.

## PROGRAM DESCRIPTION AND METHODS

**Figure 1.**
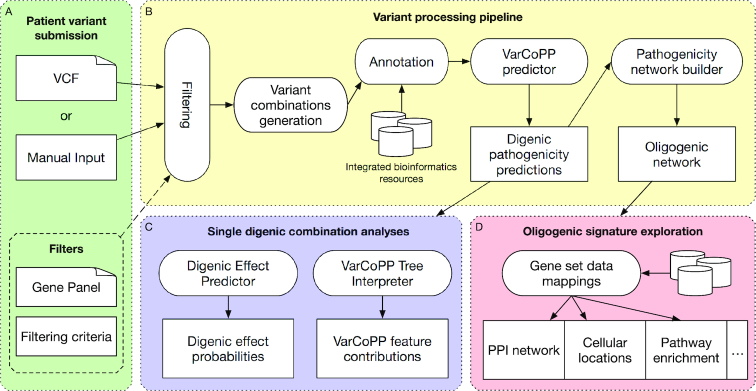
ORVAL flowchart highlighting the major components of the platform. (**A**) Users can submit variants using a Variant Call Format (VCF) file or a tab-delimited variant list. The variants can be filtered with some predefined criteria or by using a gene panel. (**B**) Once submitted, variants are processed by a pipeline first applying the selected filters, then generating all di-,tri- and tetra-allelic variant combinations and annotating them using public bioinformatics resource data, then predicting which variant combinations may be disease-causing candidates with the VarCoPP predictor. Finally, these candidate variant combinations are aggregated at the gene level to build an oligogenic network. (**C**) By selecting a specific digenic variant combination, users can run a predictor to know the DE probabilities and can get an interpretation of the VarCoPP prediction based on its features. (**D**) It is also possible to interact with the oligogenic network to filter and explore specific oligogenic signatures. A dedicated page shows how the selected gene set maps with multiple cross-references to give an insight into the biological context.

The ORVAL web platform consists of a submission form, where users can submit genetic variant data along with filtering criteria, and a variant processing pipeline that first generates variant combinations, annotates them with variant, gene and combination level information, and then predicts which variant combinations may potentially be associated with the disease. The candidate digenic predictions are then used to rank gene pairs and build an interactive oligogenic network that can be further explored. All these description levels are enhanced by known cross-references. Figure 1 summarizes the workflow and the components of ORVAL. The platform technologies are described in details in Supplementary Information (Text S1).

### Variant submission and processing

ORVAL manages its variant processing pipeline with a secure asynchronous queuing system where jobs get assigned an Universally Unique Identifier (UUID), for every submission. Users can access a job page to track the status of their submissions and bookmark them for later use. It is also possible to provide an email address to be informed when a job has been completed. The analysis results are provided via a unique link and are accessible for 7 days starting from the time of submission. No input data is stored.

The ORVAL platform accepts a list of variants from a single individual as input. These variants can be entered manually or provided in a Variant Calling File (VCF) (23) (compressed or not). Users can also choose to apply filtering options that discard variants based on a given threshold of Minor Allele Frequency (MAF), obtained from the ExAC database (24), their genomic and exonic positions or their synonymous effect (Figure 1A). Applying these filters is highly recommended to ensure that the remaining variants are in accordance with the variant types that were used to train the predictive methods integrated in ORVAL. Users can also provide a gene panel that will be used to restrict the analysis to only the genes of interest.

The ORVAL pipeline follows the processing steps described in Figure 1B. All variants are annotated based on the Ensembl GRCh37/hg19 genome version database (25) and external annotation sources necessary for the pathogenicity predictions. These annotations are: the variant CADD score (3), the protein sequence from UniProt (26), the gene recessiveness and haploinsufficiency probabilities from the dbNSFP database (27), the Gene Damage Index (GDI) that provides the susceptibility of a gene to disease (28), and the Biological Distance that shows the biological relatedness between any two genes based on PPI information (29).

After annotation, ORVAL creates all possible (i.e. bi-allelic, tri-allelic and tetra-allelic) combinations of variants occurring in gene pairs and applies the VarCoPP method (21) to predict their pathogenicity scores. Each prediction provides two predictive scores (i.e. a Support Score (SS) and a Classification Score (CS)) whose previously defined thresholds (21) determine whether a digenic combination is predicted as potentially disease-causing or neutral. These scores are also assigned confidence labels (i.e. 99% − confidence and 95% − confidence), providing a clear signal to identify the potentially most relevant pathogenic combinations.

### Digenic combination predictions

Once variants have been processed and the predictions are available, the main Results page shows, in order, the oligogenic network inferred from the predictions, summary statistics of all available gene-pairs and, finally, a detailed perspective on all predicted variant combinations that were found in the patient’s data.

**Figure 2.**
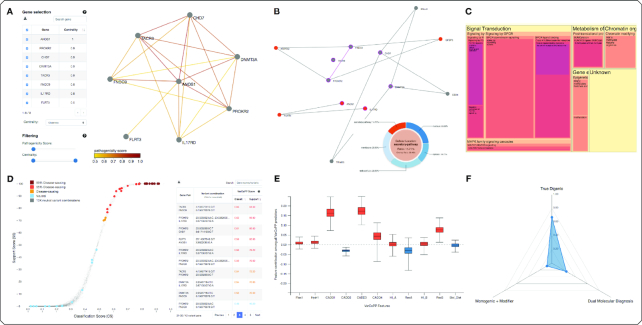
Examples of the main output figures of ORVAL. (**A**) An interactive oligogenic network built from all gene pairs having at least one predicted candidate combination. The edge are coloured based on a *pathogenicity score* (highest Classification Score (CS) for a pair). The genes can be filtered out manually or based on their centrality. The edges can be pruned based on the pathogenicity score. (**B**) A protein–protein interaction network where the central nodes circled in purple represent the proteins from a selected oligogenic module and the external nodes are the first-level interactors. Direct interactions (e.g. FNDC9-PROKR2) are coloured in purple. A pie chart showing the protein cellular locations is used to highlight the corresponding nodes in the network (here, *secretory-pathway*. (**C**) A Tree-map representing the Reactome ontology sized proportionally to the number of mapped genes from the oligogenic module and colour according to the level on the ontology hierarchy. On this example, the most represented pathways are part of the *Signal Transduction* ontology. (**D**) An S-plot representing the classification of all digenic combinations as being neutral (in blue) or potentially disease-causing (from orange to dark red) depending on the predicted VarCoPP CS and Support Score (SS). The table on the right shows the gene pair, variants and prediction scores. (**E**) A boxplot chart, displayed for a specific digenic combination, showing the contribution of each predictive features into the disease-causing class (in red) or neutral (in blue). (**F**) A spider plot, displayed for a specific digenic combination, showing the probabilities for each class of digenic effect predicted by the DE Predictor, with a highest probability for *True Digenic*.

In the latter Digenic Predictions section (bottom Results page), all digenic variant combination predictions are visualized in the form of an interactive S-plot (Figure 2D) based on the two pathogenicity scores provided by VarCoPP. Each point in the curve is a digenic variant combination whose colour represents its pathogenicity confidence (see legend in Figure 2D). A dynamic summary table next to the S-plot provides a complete list of all visualized combinations, ordered from high to low pathogenicity scores. Each combination is linked to additional detailed information.

By clicking on a digenic combination in the S-plot or the summary table, the detailed information page opens, showing the VarCoPP pathogenicity prediction, the DE prediction (provided that the combination is predicted to potentially be pathogenic) and other useful annotations specific to the selected variant combination (Figure 1C).

The Pathogenicity Prediction Information (Figure 2E) aims to explain the decision made by the predictor: it shows, by using box plots, the preference of each feature used by VarCoPP for either the positive (red colour) or the neutral (blue colour) class (21). Details on the method inferring these preferences from the predictor can be found in (30).

The DE prediction (see Figure 2F) identifies for a candidate variant combination the likelihood that it is a True Digenic, Monogenic + Modifier or a Dual Molecular Diagnosis case. A radar plot visualises these three possibilities, while a small table is also provided that shows the associated probabilities.

Biological annotations associated with the digenic combination are provided at the bottom of the detailed information page with cross-references to other bioinformatics resources. The users can get information at the gene level (e.g. gene name, Ensembl gene ID, recessiveness and haploinsufficiency probability), variant level (e.g. zygosity, dbSNP ID, allele frequency) and gene pair level (e.g. information on the biological distance of the two genes).

### Gene pair ranking

The Gene Pair Ranking panel (at the middle of the Results page) aggregates the information generated for each variant combination at the level of the gene pair, providing an insight on the pathogenicity of the gene pairs in the data. This information is displayed in a table that includes summary statistics per gene pair, such as the percentage and number of predicted candidate disease-causing variant combinations, as well as the median pathogenicity scores. The gene pairs are initially ranked according to their percentage of candidate combinations, followed by the median pathogenicity scores.

### Oligogenic signatures exploration

As genes can occur in multiple gene pairs, a network can be constructed that consists of all gene pairs that contain at least one candidate variant combination (see Figure 2A). We refer to this top level of aggregation as the *Oligogenic Network* (see top of the Results page). The colour of an edge varies depending on the highest Classification Score (CS) for the gene pair. All genes in this network are also presented in a table, along with precomputed centrality measures (31). With this table, users can remove genes from the network or they can use filters based on the centrality or the CS to restrict the analysis to the most relevant gene pairs.

Users can interact with the network: selecting a node/gene opens a side panel that shows information about the gene and about the set of genes in the same connected component, called an *Oligogenic Module* in ORVAL. This side panel shows some module-relevant metrics, such as the size, the graph density, the average pathogenicity score, as well as a summarised pathway information for the involved genes. This panel also links to a dedicated module page that contains additional information, including the associated PPIs, protein cellular locations and pathway mappings of the involved genes (Figure 1D).

The PPI network is built from the set of proteins belonging to the selected module, using the last version of the ComPPI database (32). The resulting network is visualised as an interactive circle-shaped network (Figure 2B) where the proteins from the Oligogenic Module and external proteins directly interacting with them are represented. To limit the size of the network, these external proteins are represented only if they interact with at least two proteins of the selected Oligogenic Module. The cellular location of every protein in the network is represented as an interactive pie chart that can be used to highlight the proteins from a specific location in the network. That PPI representation can assist in studying epistatic effects that could be caused by the direct and indirect interactions between proteins of the Oligogenic Module (14).

The biological pathways that are most strongly associated with the genes of the oligogenic module can be explored in the Pathway Mappings panel. This view provides a graphical pathway Tree-map (Figure 2C), i.e. a plot where boxes represent nested pathways according to their hierarchical level in the pathway ontology of Reactome (33) and whose size is determined by the number of genes they contain. Detailed information about the genes involved in each pathway level is shown in a dynamic table. This pathway mapping gives an insight on the biological phenotypes that could be affected by the genes of the selected oligogenic module, as shown in the work of An *et al.* (13).

### Results availability

Every table can be downloaded in a tab-separated values (TSV) format, while the oligogenic network (Figure 2A), module network and PPI network (Figure 2 B) can be downloaded in the GraphML format, so they can be easily imported in network analysis programs. All objects can be downloaded in the form they were originally obtained or after the application of any post-hoc filters (e.g. gene and edge filters or custom search query). The S-plot figure is available publication-ready in PNG format.

### Documentation

ORVAL has a user-friendly interface that provides guidance, at every step, on how to use the tool and interpret the results. Help buttons with summarized guidance are present in all panels of ORVAL, while warning messages provide information on how to tackle exceptional issues that may arise during data submission or exploration of results. The Documentation page of ORVAL contains a standalone in-depth guide discussing the data submission, filtering and annotation process of the user’s data, as well as of the predictive methods and the exploration of the results with case examples.

## DISCUSSION

### Examples of oligogenic support with ORVAL

To illustrate ORVAL’s relevance for geneticists and clinicians, we briefly discuss here the results for some recently published cases that are associated with diseases having high genetic and phenotypic heterogeneity and show indications of oligogenicity. These cases are completely unknown to the integrated predictive methods.

ORVAL supports the suspicions of oligogenicity for a patient with mild hypertrophic cardiomyopathy, carrying three potentially causative variants in the genes: MYH6, DSC2 and DSG2 (34). All variant combinations were predicted as candidates with high confidence, creating a trigenic oligogenic network. The integrated PPI network informed about the physical connection between proteins *DSC2* and *SSG2*, while the pathway treemap illustrated the involvement of the genes DSC2 and DSG2 in cell apoptosis, and of MYH6 in muscle contraction, further confirming that they can contribute in different phenotypes that can be blended in an individual: arrhythmogenic cardiomyopathy and hypertrophic cardiomyopathy, respectively.

Moreover ORVAL supports the oligogenic hypothesis for a patient with congenital long QT syndrome (LQTS), carrying variants in three LQTS-associated genes: KCNQ1, KCNH2 and KCNE1 (35). These genes created, again, a trigenic oligogenic network in ORVAL. We could confirm with high confidence the author suspicions for the pathogenicity of the gene pair KCNQ1 and KCNH2, as it obtained the highest median pathogenicity score. The specific combination KCNH2:p.K897T and KCNE1:p.G38SK by itself is neutral, confirming the modifier effect these two genetic variants have on the phenotype. More information on the two cases is available as Supplementary Information (Text S2, Supplementary Figures S1–S8).

### Limitations and future improvements

ORVAL offers a novel way to explore the oligogenic nature of a patient’s phenotype. Nevertheless, the interpretation of the results remains a difficult task that still requires further manual inspection. To improve interpretation, we plan to integrate the possibility to use parent (trio) variant data, so that an assessment of the inheritance pattern can also be made. Additional variant pre-filtering options will also be evaluated. The integration of the patient’s phenotypic information and its relation with other phenotypes could also offer more context to the results. We plan to explore other network-related resources, such as disease networks, as well as improve or integrate new predictive methods for oligogenic analysis. Patient cohort analyses are not yet supported, something that can be resolved with a big data pipeline that we are currently developing. Finally, the predictive quality of the methods used in ORVAL is dependent on the quality of the data in DIDA, which is noisy in the sense that not every instance has the same quality, even though they are all from peer-reviewed publications and a curating efforts were made. To improve the DIDA quality we aim to expand it but also introduce mechanisms for the community rating of its content so that high-quality subsets can be used for training and debated cases can be excluded with clear motivations.

## CONCLUSION

Research on oligogenic diseases is still at an exploratory stage due to the lack of resources to analyse variants in combinations across several loci. This task is even more difficult due to the phenotypic and genetic heterogeneity associated with cases. Nevertheless, it is essential to offer the opportunity of a combinatorial variant combination analysis with an interactive exploration of the results to derive a biological explanation for patients. ORVAL offers such an innovative web-platform that integrates different resources to predict potentially disease-causing oligogenic variant combinations and visualizes the results within their biological context. This tool provides a new essential step towards helping clinicians and researchers to improve their oligogenic investigations by formulating new hypotheses to study more complex genetic diseases.

## Supplementary Material

gkz437_Supplemental_Files
