# Supplementary material for: ORVAL: a novel platform for the prediction and exploration of disease-causing oligogenic variant combinations
Source: Nucleic Acids Res. 2019 May 31;47(W1):W93–8. doi: 10.1093/nar/gkz437 (PMC6602484; doi:10.1093/nar/gkz437)
Supplement: gkz437_Supplemental_Files [file gkz437_supplemental_files.pdf]

Supplementary Information for

ORVAL: A novel platform for the prediction and exploration of disease-causing oligogenic variant combinations

Alexandre Renaux, Sofia Papadimitriou, Nassim Versbraegen, Charlotte Nachtegael, Simon Boutry, Ann Nowé, Guillaume Smits, Tom Lenaerts

Corresponding authors:
Tom Lenaerts: tlenaert@ulb.ac.be

This PDF file includes:
Supplementary Text S1 and S2
Supplementary Figures S1 to S8
References for SI reference citations

Supplementary Text S1: Supplementary information on the technologies used to implement ORVAL

This web platform has been built in *Python* using the *Django framework* (https://www.djangoproject.com) and is running on an *Apache HTTP Server* (https://httpd.apache.org). Machine learning based predictors are built with *scikit-learn* (https://scikit-learn.org/) and *Numpy* (http://www.numpy.org). The VarCoPP predictions are decomposed into feature contributions by the *treeinterpreter* package (https://github.com/andosa/treeinterpreter). Network computations are based on *NetworkX* (https://networkx.github.io).

The client-side user interface has been designed with the *Bootstrap* framework (https://getbootstrap.com), the *JQuery* library (https://jquery.com). The interactive charts and tables have been built using *D3.js* (https://d3js.org) and *DataTables* (https://datatables.net). Icons are taken from *Font Awesome* (https://fontawesome.com) and the documentation tooltips are displayed with *Popper.JS* (https://popper.js.org). Chromosome visualizations are developed with Ideogram.JS (https://eweitz.github.io/ideogram/). All developed JavaScript code is compiled and bundled with *Babel* (https://babeljs.io) and *Webpack* (https://webpack.js.org).

External annotation data and analysis results are securely stored in a *PostgreSQL* database (https://www.postgresql.org). Analysis tasks are all running asynchronously and are handled by *Celery* (http://www.celeryproject.org), a distributed task queuing system, coupled with the message broker *RabbitMQ* (https://www.rabbitmq.com), to manage computational resources and job status.

ORVAL has been tested with major modern browsers such as Google Chrome (71+), Mozilla Firefox (64+), Safari (12+) and Microsoft Edge (44+).

Supplementary Text S2: Supplementary information for the examples having oligogenic support with ORVAL

We decided to explore the potentials of ORVAL in supporting oligogenic disease signatures, or confirming the neutrality of asymptomatic patients, in recent pedigree cases that were published in the scientific literature that involve diseases with high genetic and phenotypic heterogeneity that show oligogenic potentials.

The **first published case** concerned a 49-year old patient with mild symmetric cardiac hypertrophy(1). The authors, after a variant prioritization procedure, found three potentially causative variants in the related genes: MYH6:c.2292+2T>C, DSC2:c.2688_2688delinsGAA and DSG2:c.877A>G (in homozygous state). The MYH6 mutation has been linked to the biventricular hypertrophic phenotype, while the DSC2 variant has been known to affect the phenotypic expression of ARVC/D mutations. On the other hand, the homozygous DSG2 variant represents a major criterion for arrhythmogenic cardiomyopathy.

ORVAL supports the conclusions drawn for this patient, by predicting disease-causing digenic combinations between all pairs of this gene trio and creating a potential pathogenic oligogenic network (**Figure S1**). The gene pair DSC2 and DSG2 carried the most pathogenic disease-causing combination (**Figure S2**). An interesting aspect of the analysis was the fact that the digenic combination between the genes MYH6 and DSG2, each one carrying a mutation associated with the hypertrophic phenotype and arrhythmogenic cardiomyopathy, respectively, was predicted as a Dual Molecular Diagnosis case, supporting a blending phenotype. The rest of the combinations were predicted as True Digenic. All three resulting proteins share at least the cytosol cellular location, although the protein *MYH6* does not interact directly or indirectly (with one degree of separation) with the other two proteins (**Figure S3**). This distinction is also shown in the pathway enrichment panel for that module, which shows that MYH6 is involved in regulating muscle contraction, while DSG2 and DSC2 genes are involved in cell apoptosis and cornification of keratinocytes (**Figure S4**).

The predictive capacity of ORVAL was also shown for the case of the patient's asymptomatic son, who carried the variants MYH6:c.2292+2T>C and DSG2:c.877A>G, both in heterozygous state this time. ORVAL predicted this combination as neutral, further supporting the pathogenicity of the homozygous state of the DSG2:c.877A>G and its association with arrhythmogenic cardiomyopathy.

The **second case** concerned a family associated with congenital Long-QT (LQTS) syndrome, involving clinically asymptomatic parents and three children with prolonged QT intervals and extreme bradycardia(2). The authors found four potentially causative variants in three different

LQTS-associated genes: KCNQ1:p.R583H, KCNH2:p.C108Y and p.K897T and KCNE1:p.G38S. Based on *in silico* analysis, the pedigree information and the recent literature the authors focused on the variants KCNQ1:p.R583H and KCNH2:p.C108Y, concluding that the KCNQ1:p.R583H was not associated with a severe functional impairment, whereas KCNH2:p.C108Y, a novel variant, encoded a non-functional channel that exerts dominant-negative effects on the wild-type. KCNH2:p.K897T and KCNE1:p.G38S are associated with severe phenotypes when combined with other disease-causing variants (probably with the KCNH2:p.C108Y), therefore their role is suggested to be that of a modifier.

ORVAL provided oligogenic support for the **proband II-1** of the family that contained all four heterozygous variants and was associated with a severe phenotype, having experienced two syncope episodes and continuous prolonged QT-intervals after treatment. All gene pairs of the network contained disease-causing combinations forming a predicted disease-causing oligogenic network, with the gene pair KCNQ1 - KCNH2 having the highest median pathogenicity (**Figure S5**). Looking specifically at the VarCoPP scores of the combinations, we could confirm the author suspicions for the pathogenicity of the variant combination KCNQ1:p.R583H and KCNH2:p.C108Y, as it obtained the highest pathogenicity score (**Figure S6**). The addition of the modifier KCNH2:p.K897T variant in that combination does not seem to increase the pathogenicity score. We could also support the role of the common KCNH2:p.K897T and KCNE1:p.G38SK as rather mutual modifiers or not-causative variants when present alone, as a combination of those two is predicted as neutral and that gene pair in by itself had the lowest median pathogenicity score (**Figures S5** and **S6**).

The vast majority of predicted as pathogenic digenic combinations were associated with a True Digenic digenic effect (DE). The variant combination KCNE1:p.G38S and KCNQ1:p.R583H was predicted as Dual Molecular Diagnosis, a questionable finding, although the probability of that class was very close to the probability of Monogenic + Modifier DE class with 0.382 and 0.319 probability, respectively.

Even though the pair KCNH2 - KCNQ1 was predicted to contain a higher pathogenicity score, we could understand the role of the KCNH1 common variant as a potential modifier in the severity of the symptoms for the patient, as the *KCNH1* protein is the one that connects the two genes with an indirect protein-protein interaction (**Figure S7**). This gene is also implicated in the same biological processes as KCNH2 and KCNQ1, e.g. it is involved in cardiac conduction, as well as the regulation of the membrane potassium channels, findings that ORVAL can support as well in the Pathway Mapping section. (**Figure S8**).

Supplementary Figures

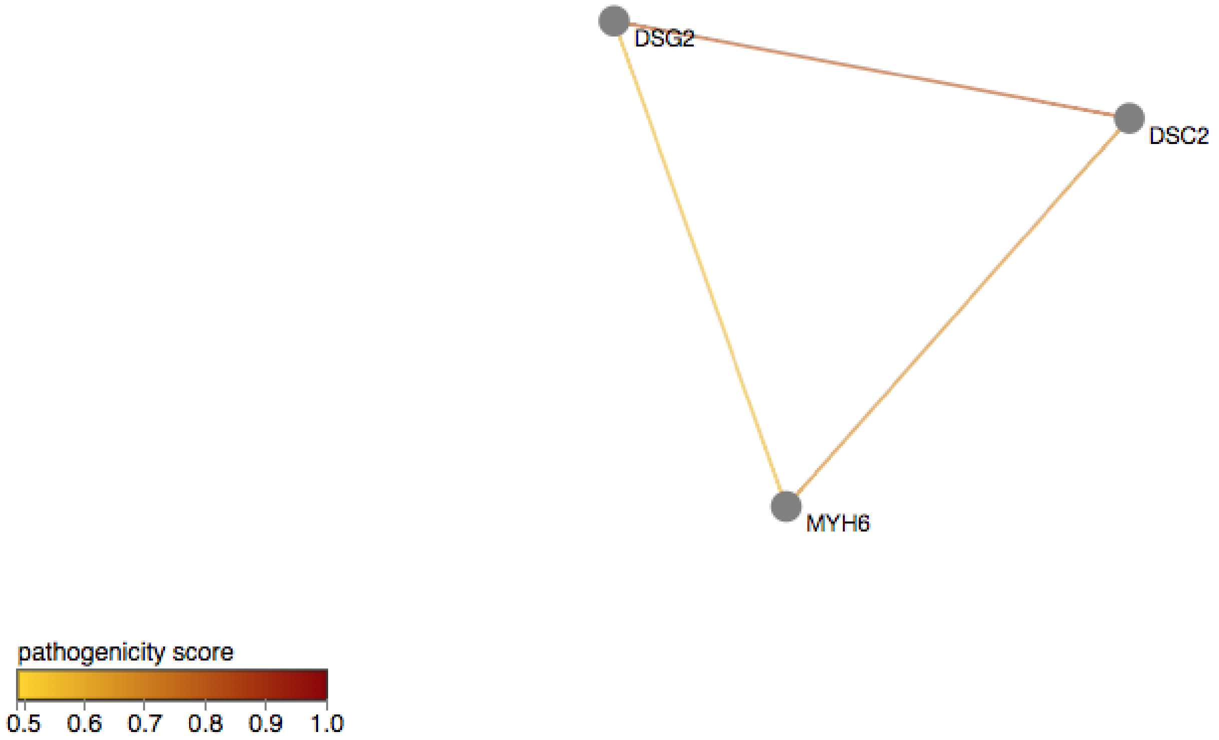

Figure S1. The predicted oligogenic network for patient associated with mild hypertrophic cardiomyopathy syndrome. The gene pair DSG2 - DSC2 has a higher pathogenicity score based on the variant combination predictions by VarCoPP for that pair, as depicted by the darker edge colour.

Search: Gene names/variants

Gene Pair	Variant combination (Click for more details)	VarCoPP Score	
		Classif.	Support
DSC2<br>DSG2	18:28648000:-:TC<br>18:29104714:A:G	0.81	100.00
DSC2<br>MYH6	18:28648000:-:TC<br>14:23865901:A:G	0.69	99.40
DSG2<br>MYH6	18:29104714:A:G<br>14:23865901:A:G	0.57	85.60

1-3 / 3 variant pairs

Previous 1 Next

Figure S2. The summary table with the VarCoPP predictions for all variant combinations present in the patient associated with mild hypertrophic cardiomyopathy. All combinations were predicted as disease-causing.

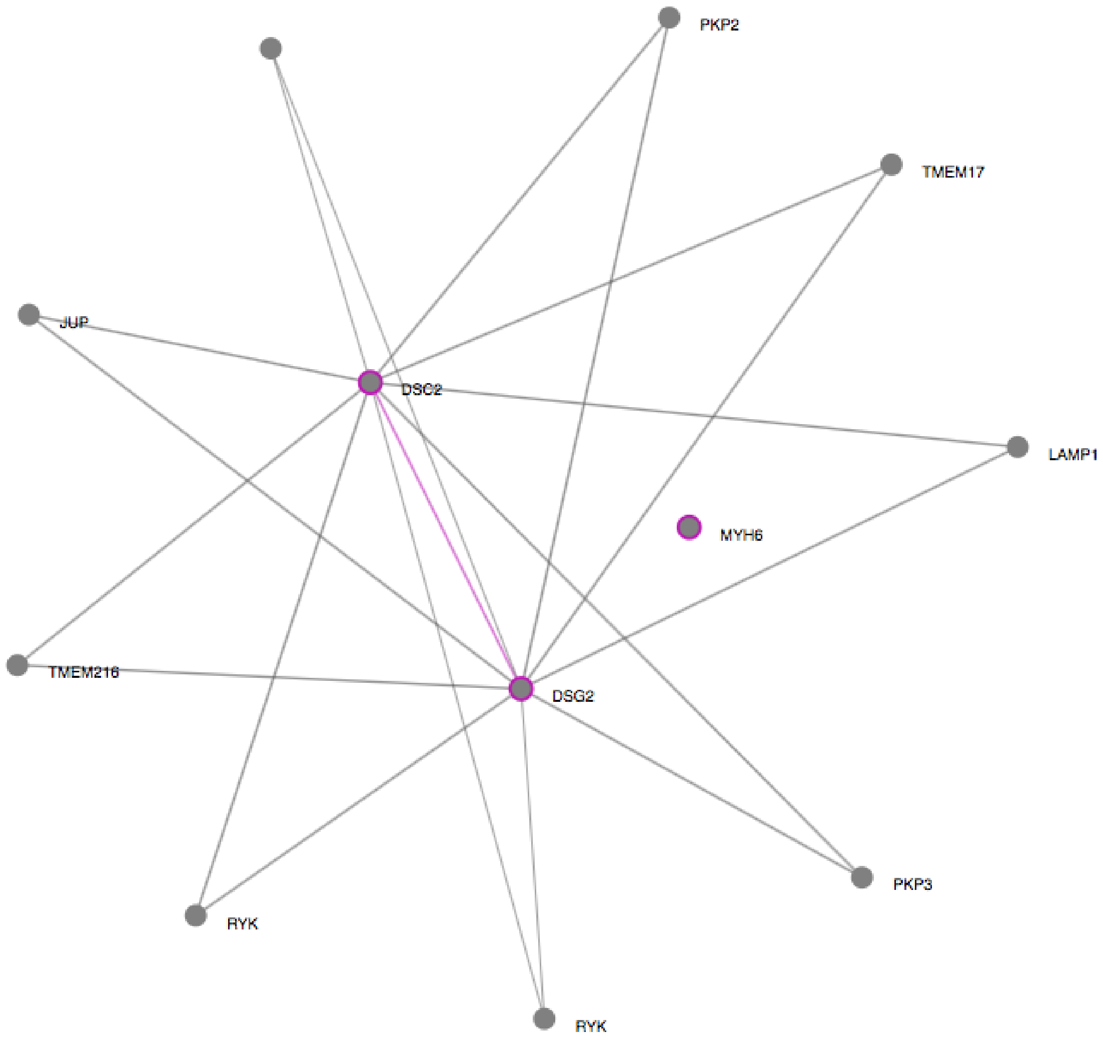

Figure S3. The protein-protein interaction (PPI) provided at the oligogenic module page for the patient associated with mild hypertrophic cardiomyopathy, containing the first-level interactors of the module proteins. *DSC2* and *DSG2* are directly interacting, while *MYH6* does not share any first-level interactors with those proteins.

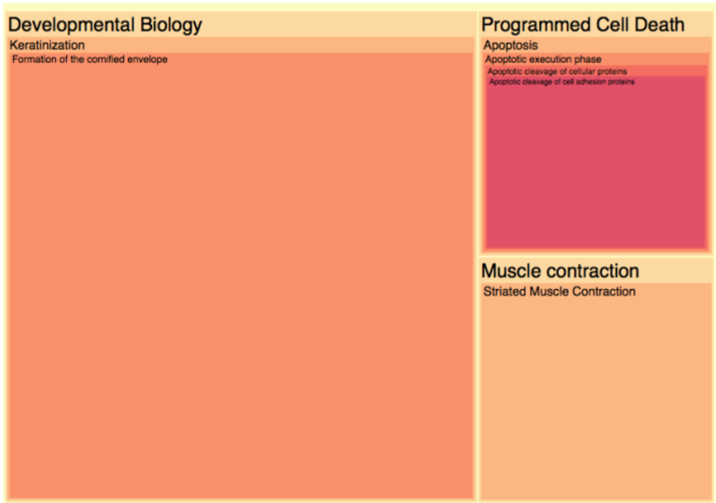

Search: Gene / Pathway

Pathway	Genes
Developmental Biology	DSC2,DSG2
Keratinization	DSC2,DSG2
Formation of the cornified envelope	DSC2,DSG2
Programmed Cell Death	DSG2
Apoptosis	DSG2
Apoptotic execution phase	DSG2
Apoptotic cleavage of cellular proteins	DSG2
Apoptotic cleavage of cell adhesion protei...	DSG2
Muscle contraction	MYH6
Striated Muscle Contraction	MYH6

1-10 / 11

Figure S4. The pathway mappings of the three genes involved in the oligogenic network of the patient involved in mild hypertrophic cardiomyopathy.

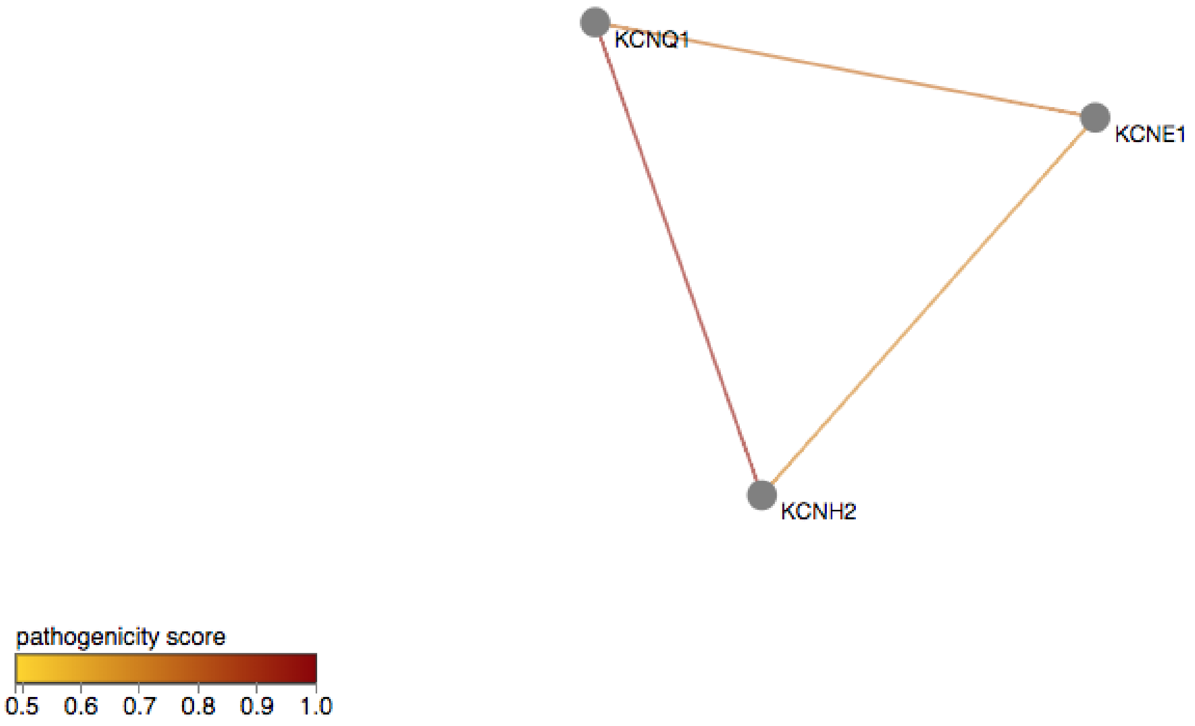

Figure S5. The predicted oligogenic network for the proband II-1 associated with congenital Long-QT (LQTS) syndrome. The gene pair KCNQ1 - KCNH2 has a higher pathogenicity score based on the variant combination predictions by VarCoPP for that pair, as depicted by the red edge colour.

Search: Gene names/variants

Gene Pair	Variant combination (Click for more details)	VarCoPP Score	
		Classif.	Support
KCNQ1 KCNH2	11:2799221:G:A 7:150656809:C:T	0.96	100.00
KCNQ1 KCNH2	11:2799221:G:A 7:150656809:C:T, 7:150645534:...	0.95	100.00
KCNE1 KCNQ1	21:35821821:T:C 11:2799221:G:A	0.76	100.00
KCNQ1 KCNH2	11:2799221:G:A 7:150645534:T:G	0.71	99.60
KCNE1 KCNH2	21:35821821:T:C 7:150656809:C:T, 7:150645534:...	0.70	98.40
KCNE1 KCNH2	21:35821821:T:C 7:150656809:C:T	0.69	97.80
KCNE1 KCNH2	21:35821821:T:C 7:150645534:T:G	0.25	0.20

1-7 / 7 variant pairs

Previous 1 Next

Figure S6. The summary table with the VarCoPP predictions for all variant combinations (including tri-allelic combinations) present in proband-II associated with congenital Long-QT (LQTS) syndrome. The combination listed first with the highest pathogenicity score contains the variants KCNQ1-p.R583H and KCNH2-p.C108Y that were mostly studied in the paper.

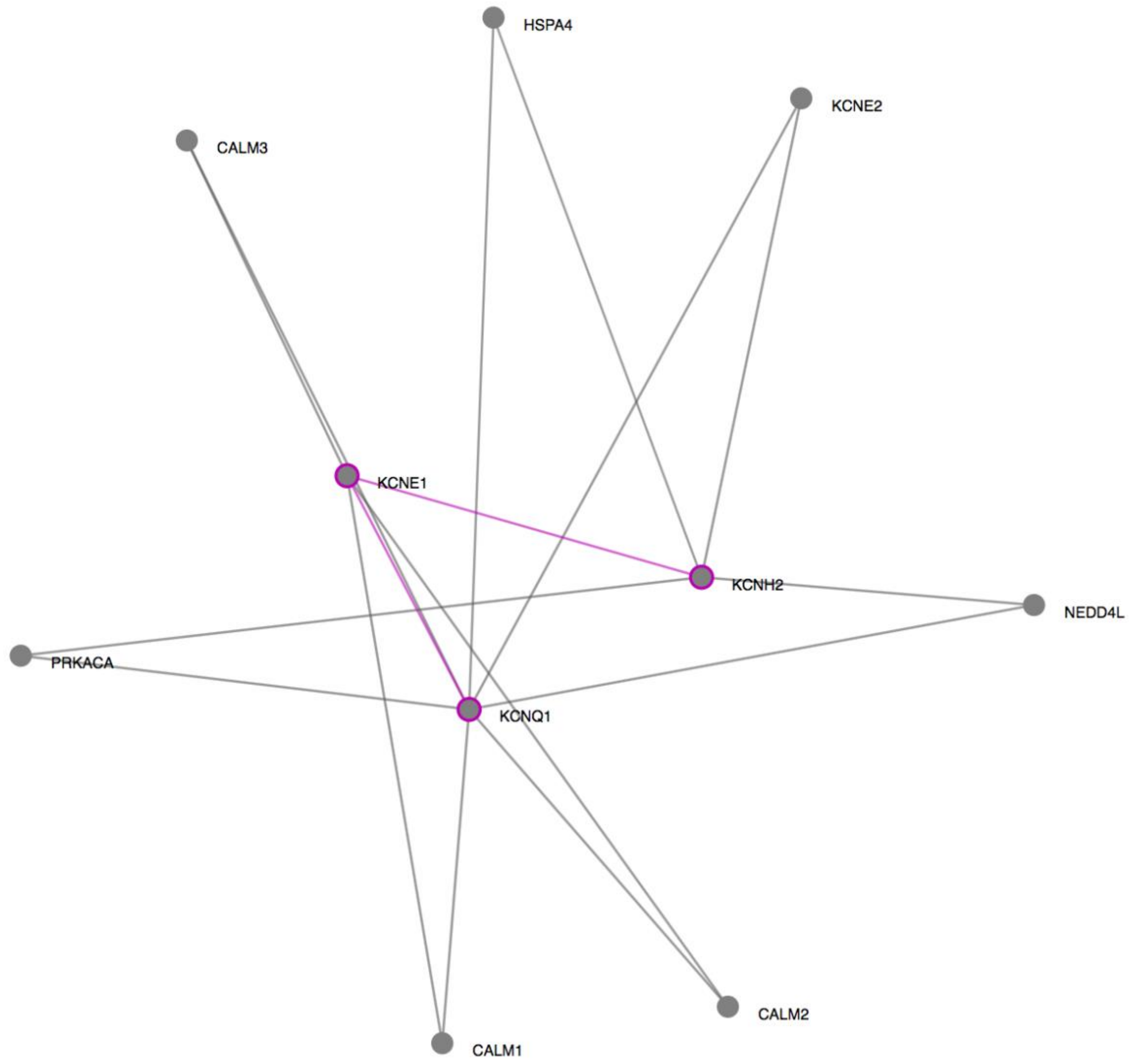

Figure S7. The protein-protein interaction (PPI) provided at the oligogenic module page for the LQTS-associated patient, containing the first-level interactors of the module proteins. *KCNE1* connects with an indirect interaction the genes *KCNH2* and *KCNQ1*.

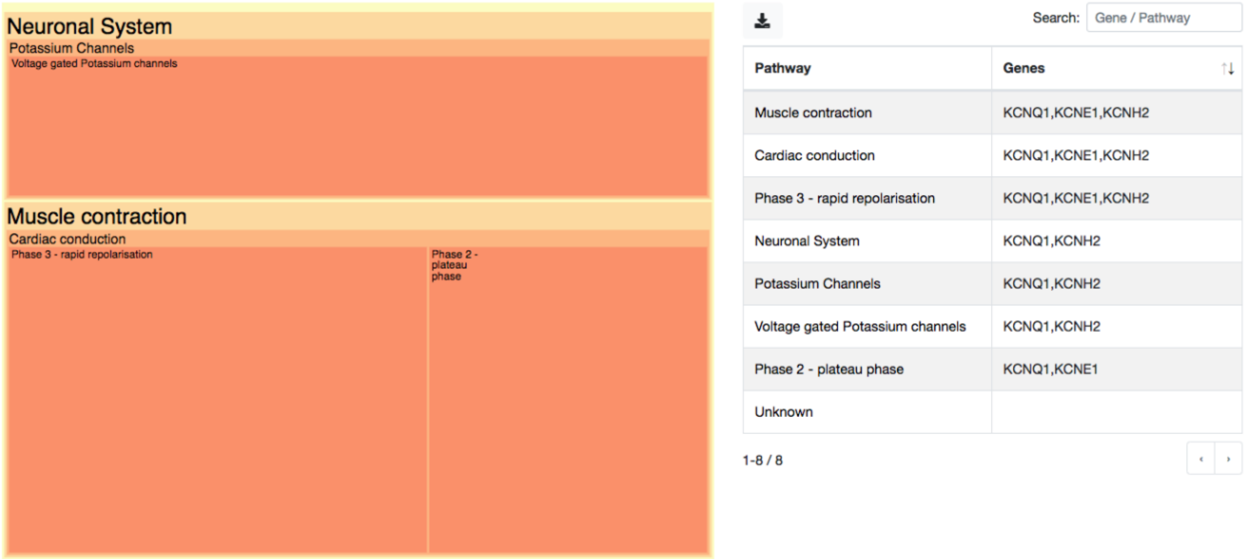

Pathway	Genes
Muscle contraction	KCNQ1,KCNE1,KCNH2
Cardiac conduction	KCNQ1,KCNE1,KCNH2
Phase 3 - rapid repolarisation	KCNQ1,KCNE1,KCNH2
Neuronal System	KCNQ1,KCNH2
Potassium Channels	KCNQ1,KCNH2
Voltage gated Potassium channels	KCNQ1,KCNH2
Phase 2 - plateau phase	KCNQ1,KCNE1
Unknown	

Figure S8. The pathway mappings of the three genes involved in the oligogenic network of proband-II. All of them are regulating the muscle, and more specifically cardiac, contraction, and the flow of potassium in the cellular membrane channels.

Supplementary references

1. Castellana,S., Mastroianno,S., Palumbo,P., Palumbo,O., Biagini,T., Leone,M.P., De Luca,G., Potenza,D.R., Amico,C.M., Mazza,T., *et al.* (2019) Sudden death in mild hypertrophic cardiomyopathy with compound DSG2/DSC2/MYH6 mutations: Revisiting phenotype after genetic assessment in a master runner athlete. *J. Electrocardiol.*, **53**, 95–99.

2. Zullo,A., Frisso,G., Detta,N., Sarubbi,B., Romeo,E., Cordella,A., Vanoye,C.G., Calabrò,R., George,A.L. and Salvatore,F. (2017) Allelic Complexity in Long QT Syndrome: A Family-Case Study. *Int. J. Mol. Sci.*, **18**.
